# Vitreous metastasis from cutaneous melanoma: diagnosis and management

**DOI:** 10.5935/0004-2749.2022-0215

**Published:** 2023-04-17

**Authors:** Noy Ashkenazy, J. William Harbour, Sander R. Dubovy, Thomas A. Albini, Jayanth Sridhar, Nish Patel, Eric D. Hansen, Eduardo Uchiyama, Patrick E. Rubsamen, Zelia M. Correa

**Affiliations:** 1. Bascom Palmer Eye Institute, University of Miami Miller School of Medicine, Miami, Florida; 2. University of Texas Southwestern Medical Center, Department of Ophthalmology, Dallas, Texas; 3. Sylvester Comprehensive Cancer Center, University of Miami Miller School of Medicine, Miami, Florida; 4. Florida Lions Ocular Pathology Laboratory, University of Miami Miller School of Medicine, Miami, Florida; 5. Henry Ford Hospital, Department of Ophthalmology, Detroit, Michigan; 6. John A. Moran Eye Institute, University of Utah, Salt Lake City, Utah; 7. Retina Group of Florida, Fort Lauderdale, Florida

**Keywords:** Melanoma, Eye neoplasms, Skin neoplasms, Neoplasm metastasis, Vitreous body, Immune checkpoint inhibitors, Immunotherapy, Intravitreal injections, Melphalan, Methotrexate, Melanoma, Neoplasias oculares, Neoplasias cutâneas, Corpo vítreo, Metástase neoplásica, Inibidores de checkpoint imunológico, Imunoterapia, Injeções intravítreas, Melfalan, Metotrexato

## Abstract

**Purpose:**

To report the clinical findings, treatments, and outcomes in a series of patients with vitreous metastasis from cutaneous melanoma.

**Methods:**

This single-center, retrospective, interventional case series included patients with biopsy-confirmed vitreous metastasis from cutaneous melanoma diagnosed between 1997 and 2020. Standard 23- or 25-gauge pars plana vitrectomy was performed for diagnostic sampling. Sclerotomies were treated with double or triple freeze-thaw cryotherapy. Perioperative intravitreal injections of melphalan (32 μg/0.075 mL) were administered, when indicated. Visual acuity, intraocular pressure, and systemic and ocular treatment responses were reported.

**Results:**

Five eyes of five patients with unilateral vitreous metastasis from cutaneous melanoma were identified. The median age at diagnosis was 84 (range, 37-88) years. The median follow-up after ophthalmic diagnosis was 28 (8.5-36) months; one patient did not have a follow-up. The initial visual acuity ranged from 20/30 to hand motions. Baseline clinical findings included pigmented or non-pigmented cellular infiltration of the vitreous (5/5), anterior segment (4/5), and retina (3/5). Four patients had secondary glaucoma. Systemic therapy included checkpoint inhibitor immunotherapy (n=3, all with partial/complete response), systemic chemotherapy (n=2), surgical resection (n=3), and radiation (n=2). The median time from primary diagnosis to vitreous metastasis was 2 (2–15) years. One patient had an active systemic disease at the time of vitreous metastasis. The final visual acuity ranged from 20/40 to no light perception. Ophthalmic treatment included vitrectomy in all five patients, intravitreal administration of melphalan in three, and intravitreal administration of methotrexate in one. One patient required enucleation, and histopathology revealed extensive invasion by melanoma cells.

**Conclusions:**

Vitreous metastasis from cutaneous melanoma can present as a diffuse infiltration of pigmented or non-pigmented cells into the vitreous and may be misdiagnosed as uveitis. Diagnostic pars plana vitrectomy and periodic intravitreal chemotherapy may be indicated.

## RESUMO

**Objetivo:**

Descrever os achados clínicos, tratamentos, e desfechos em uma série de pacientes com metástases vítreas de melanoma cutâneo.

**Métodos:**

Série retrospectiva de casos de único centra com intervenção. Pacientes incluídos tiveram seu diagnóstico de MVMC confirmado por biópsia entre 1997 e 2020. Vitrectomia via pars plana com 23 ou 25 gauge foram realizadas para obter espécimens. Esclerotomias foram tratadas com crioterapia em duplo ou triplo congelamento. Injeção intravítrea perioperatória de melfalano (32 ug/0,075 mL) foi administrada quando necessário. Foram relatados acuidade visual, pressão intraocular, resposta terapêutica sistêmica e ocular.

**Resultados:**

Cinco olhos de 5 pacientes com metástases vítreas de melanoma cutâneo unilateral foram identificados. Idade média de diagnóstico foi 84 anos (variando de 37-88). Seguimento médio após diagnóstico oftalmológico foi 28 (8,5-36) meses; 1 paciente nño teve acompanhamento. Acuidade visual inicial variou de 20/30 a movimentos de mño. Achados clínicos iniciais incluíram infiltração de células pigmentadas e não-pigmentadas no vítreo (5/5), segmento anterior (4/5), e retina (3/5). Quatro pacientes tiveram glaucoma secundário. Tratamento sistêmico incluiu imunoterapia com inibidores da via de sinalização (3 - todos com resposta parcial/completa), quimioterapia sistêmica (2), ressecção cirúrgica (3), e irradiação (2). Intervalo médio entre diagnóstico primário e metástases vítreas foi 2 (2–15) anos. Um paciente teve doença sistêmica ativa simultânea as metástases vítreas. Acuidade visual final variou entre 20/40 e SPL. Tratamento oftalmológico incluiu vitrectomia nos 5 pacientes, melfalano intravítreo em 3 e metotrexato intravítreo em 1. Um paciente precisou de enucleação. A histopatologia revelou invasão celular extensa de melanoma.

**Conclusões:**

Metástases vítreas de melanoma cutâneo pode se manifestar como uma infiltração difusa de células pigmentadas e não-pigmentadas no vítreo e erroneamente diagnosticada como uveites. Vitrectomia diagnóstica e quimioterapia intravítrea periódica podem estar indicadas.

## INTRODUCTION

Vitreous metastasis is a rare but well-described manifestation of cutaneous melanoma (CM) that typically presents as an infiltration of the vitreous by golden or brown pigment cells^(1–5)^. Vitreous metastasis of CM (VMCM) can masquerade as vitreous hemorrhage, uveitis, and endophthalmitis. With the improved survival of patients with metastatic CM, vitreous metastasis may be more common^(6)^.

The era of checkpoint inhibitor (CPI) therapy began in 2011, when the FDA approved ipilimumab (Yervoy^®^, Bristol-Myers Squibb, NY, USA), a monoclonal antibody targeting cytotoxic T-lymphocyte-associated protein 4 (CTLA-4) for unresectable or metastatic CM^(6)^. CTLA-4 resides on inhibitory CD4+ T-cells and typically suppresses immune response through the FOXP3 and TGF-β1 pathways. Its inhibition prevents interaction with antigen-presenting cells and effector T-cells, leading to immune activation^(7)^. Monoclonal antibodies against programmed death-1 (PD-1), namely, pembrolizumab (Keytruda^®^, NJ, Merck) and nivolumab (Opdivo^®^, Bristol-Myers Squibb), were approved in 2014^(6)^. PD-1 on immune cells controls intrinsic unresponsiveness of effector T-cells by attenuating antigen-specific signals. Antibodies against PD-1 limit its interaction with PD ligands 1 and 2 on tumor cells, resulting in immune activation against tumors^(7)^.

CPIs penetrate the blood-brain barrier when treating central nervous system (CNS) CM metastases^(8–10)^. The 5-year survival and progression-free survival on combination immunotherapy have increased to 52% and 36%, respectively^(6)^. CPIs are also used in renal cell carcinoma and non-small cell lung cancer^(11)^. CPI applications are evolving for other cancers. Durante et al. reported *LAG3* as a potential therapeutic target for metastatic primary uveal melanoma (PUM)^(12)^.

The survival rate in stage IV CM was historically 22%^(13)^. CNS metastases occur in up to half of the patients with VMCM and portend poorer prognoses. Systemic imaging is critical because VMCM may precede CNS metastasis in a third of patients ^(1,5)^. CPIs combined with *B-Raf* inhibitors (vemurafenib, encorafenib, and dabrafenib) and *MEK* inhibitors (trametinib and cobimetinib) may further improve survival^(6)^.

CPIs have become the first-line treatment of advanced CM and may contribute to increased reports of intraocular metastasis of CM^(9)^. Herein, we describe the features, management, and outcomes of five patients with unilateral VMCM.

## METHODS

This retrospective, consecutive, interventional case series was approved by the Institutional Review Board at the University of Miami Miller School of Medicine and was conducted after the approval of Human Subjects Committee. The research protocol adhered to the tenets of the Declaration of Helsinki and was compliant with the Health Insurance Portability and Accountability Act.

Available clinical records were reviewed. The digital database of the Florida Lions Ocular Pathology Laboratory was reviewed, including all ocular pathology reports from December 1997 to December 2019 at a single tertiary center (Bascom Palmer Eye Institute, University of Miami). VMCM diagnosed by vitreous biopsy were selected, excluding PUM. Charts were reviewed for patient demographics, cancer history/treatment, ocular treatments, and visual acuity (VA) outcomes.

All patients were initially managed with standard 23-or 25-gauge pars plana vitrectomy (PPV) for diagnostic sampling using a wide-angle viewing system, valved trocar cannulas, and localized conjunctival peritomies at the sclerotomy sites. Meticulous vitreous removal and operative steps were undertaken to ensure the integrity of the posterior segment anatomy. Vitreous specimens were sent for expert cytopathological analysis, and immunohistochemistry was done on hematoxylin-eosin (H&E) and Papanicolaou (PAP)-stained specimens when necessary to confirm the diagnosis. Sclerotomies were sutured and treated with double or triple freeze-thaw cryotherapy to reduce the risk of seeding tumor cells^(14)^. Intravitreal chemotherapy was administered, when indicated.

## RESULTS

Clinical characteristics are summarized in tables 1 and 2. Detailed history and findings are described.

### Case 1

**Figure 1.**
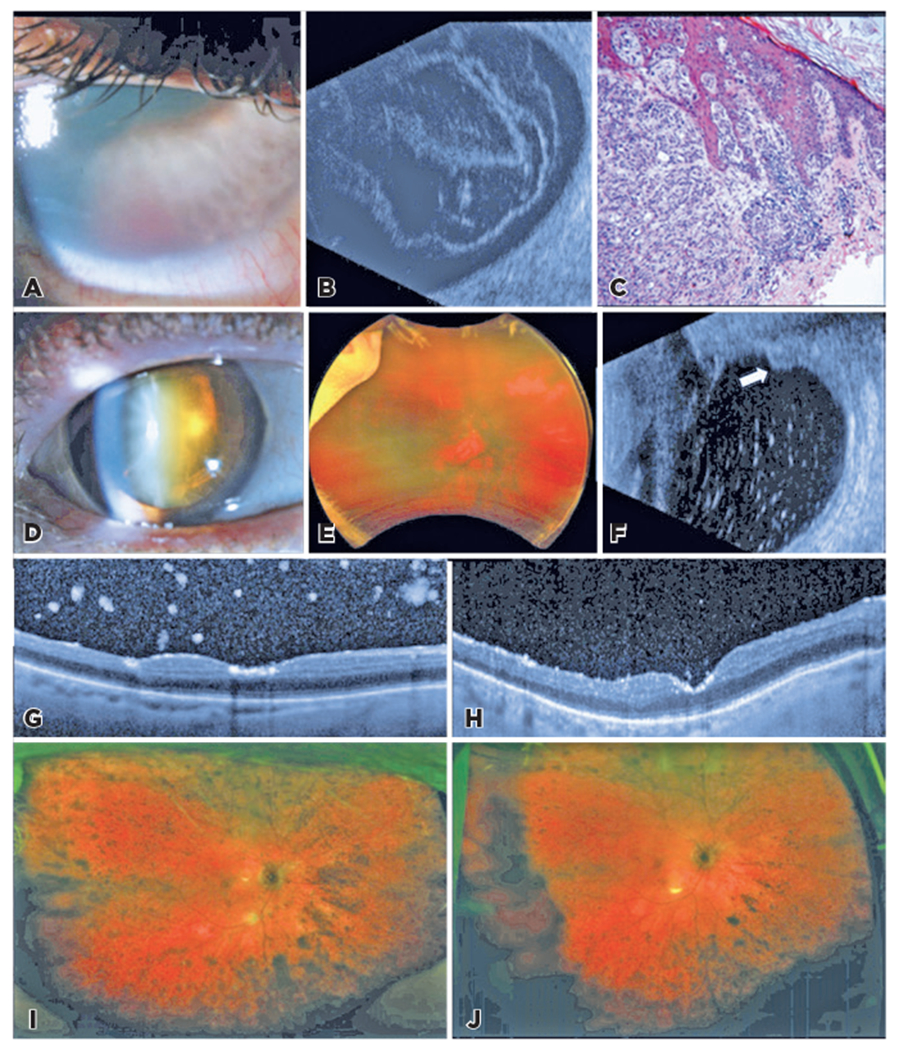
Clinical findings of cases 1 and 5 masquerading as neovascular complications. Neovascular glaucoma in case 1. (A) Slit lamp photograph shows corneal edema, rubeosis, and hyphema. (B) B-scan with diffuse vitreous opacities with membrane formation without masses. (C) Hematoxylin–eosin staining of the cutaneous shoulder biopsy demonstrates atypical, pigmented cells with prominent nucleoli, corresponding with similar cells on vitreous cytology. Apparent non-clearing vitreous hemorrhage in case 5. (D) Slit lamp photograph at presentation, with pigmentary deposits on the posterior lens surface of the cataract. (E) Wide-field fundus photograph showed hazy media and diffuse pigmentary deposition in the posterior pole. (F) B-scan illustrated numerous vitreous opacities and clumping along the residual vitreous skirt (arrow) in this patient who was previously vitrectomized. OCT 1 month after the first intravitreal melphalan (G) and following two additional monthly injections (H) suggested a reduced metastatic tumor burden in the vitreous and on the retinal surface. A corresponding reduction in the pigmentation on wide-field fundus photographs were seen at these intervals (I, J).

A 37-year-old man presented with sudden-onset vision loss and pain in the right eye for 2 days. The uncorrected VA was 20/100 and 20/50, and the intraocular pressures (IOPs) were 22 and 8 mmHg in the right and left eyes, respectively. The right pupil was poorly reactive, and slit lamp examination revealed corneal haze, a small hyphema, posterior synechiae, and rubeosis iridis (Figure 1A). The view was limited for a fundus examination. B-scan showed vitreous opacities with a membrane and absence of masses (Figure 1B). Anterior chamber paracentesis and intravitreal injection of bevacizumab 1.25 mg/0.05 mL (Avastin^®^, Genentech, CA, USA) were performed in the right eye. Pressurelowering drops were started. The left eye examination was unremarkable.

History revealed biopsy-proven stage II T4bN0M0 CM of the right shoulder with superficial spreading, with late metastasis to the chest wall that occurred 2 years ago. The treatment of the primary tumor involved resection, systemic interferon, and six sessions of external beam radiation therapy, for a total of 3600 cGy to the shoulder and axilla. Positron emission tomography/computed tomography (PET/CT) was negative in the months preceding his ocular symptoms. His right eye was managed by phacoemulsification with intraocular lens implantation, 23-gauge PPV, vitreous biopsy, and intravitreal injection of triamcinolone. No discrete tumors were noted intraoperatively. Cotton wool spots and intraretinal hemorrhages were consistent with a central retinal vein occlusion. Vitreous biopsy was consistent with VMCM, corresponding to a previous cutaneous biopsy (Figure 1C). Follow-up data was unavailable.

### Case 2

**Figure 2.**
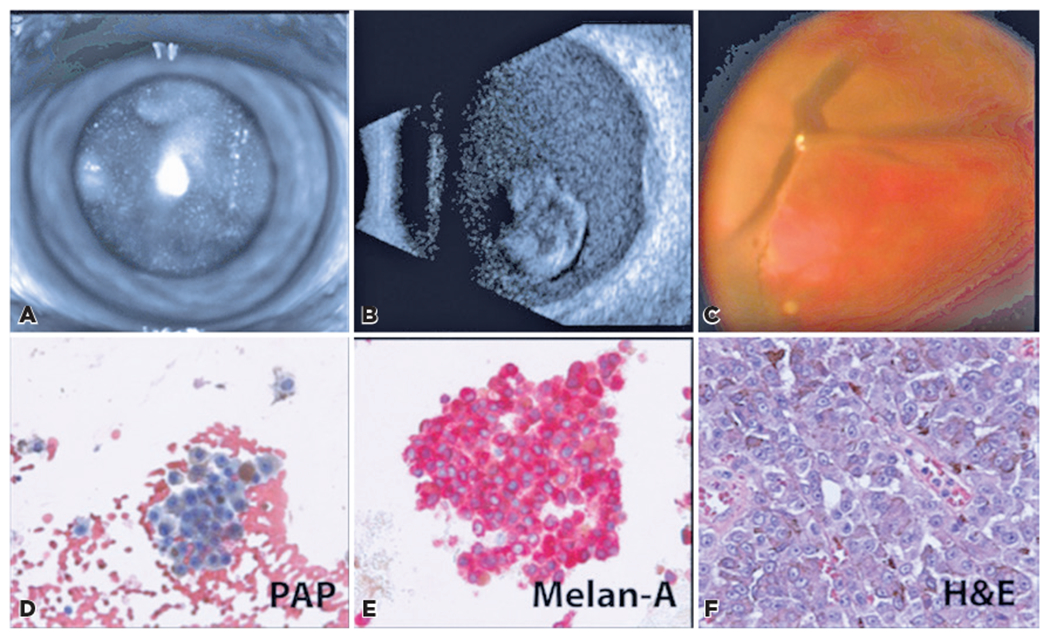
Clinical findings of case 2 masquerading as a posterior pigmented mass. (A) Posterior lenticular opacities. (B) B-scan shows dense vitreous cellularity, posterior vitreous detachment, and a hyperechoic lesion of minimal vascularity with biconvex cross-sectional shape, measuring 7.2 × 7.2 × 1.3 mm. (C) Intraoperative membrane peeling during diagnostic vitrectomy. Vitreous biopsy (D, E) showed atypical, pigmented cells with prominent nucleoli, in a background of erythrocytes. Cutaneous biopsy results from the original cheek lesion (F) are consistent with melanoma. No magnifications are available.

An 87-year-old man presented with decreased vision and floaters for 3 months in the left eye, worsening during the past month. His health history included stage 4 head and neck melanoma secondary to a cheek lesion, for which the patient underwent Mohs resection and received nivolumab with complete response (last dose was 3 weeks ago). The uncorrected VA was 20/400 in the right eye and hand motions in the left eye. The IOP was 13 mmHg bilaterally. A slit lamp examination of the left eye revealed a nuclear cataract with posterior capsular pigment (Figure 2A). The fundus was poorly visible. B-scan ultrasonography showed diffuse, mobile subhyaloid opacities with a vascularized lesion at 9:30 that measured 7.5 mm in diameter and 1.3 mm in thickness (Figure 2B). The patient underwent PPV, vitreous biopsy, membrane peel, endolaser, air-fluid exchange, and triple freeze-thaw sclerotomy closure of the left eye. Intraoperatively, an elevated choroidal mass nasal to the optic nerve, an epiretinal membrane layered with brown pigment, and a superior retinal break were found (Figure 2C). Cytology confirmed the diagnosis of VMCM (Figure 2D–E), corresponding to the original histological specimen of the CM lesion (Figure 2F).

**Figure 3.**
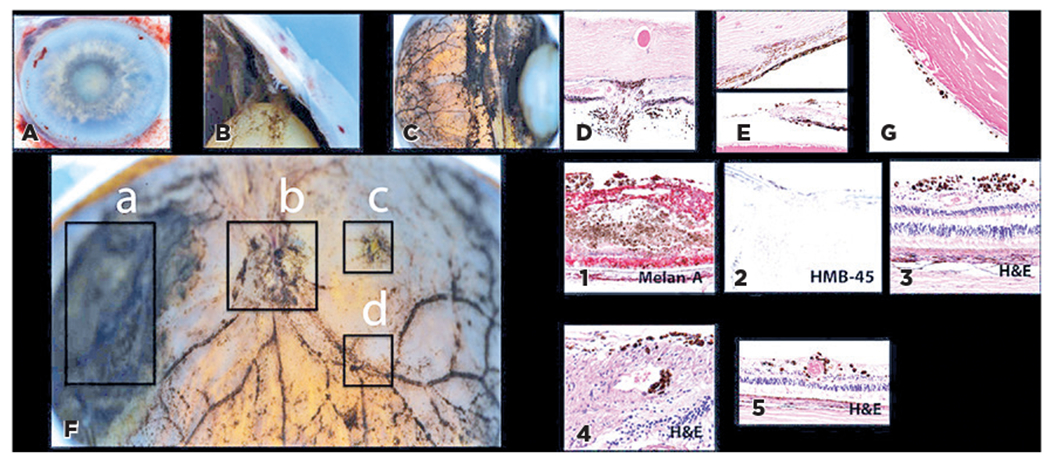
Case 2. Gross enucleation specimen photographs (A–C) and histology slides (D–G). Gross specimens with extensive deposition of pigmented melanoma cells invading the iris, (A) ciliary body and angle (B), and posterior segment (C). The corresponding hematoxylin–eosin stains demonstrate invasion of the sclerotomy suture track (D, original magnification, ×200), trabecular meshwork (E, original magnification, ×200), iris surfaces (F, original magnification, ×100), and posterior lens capsule (G, original magnification, ×200). Coronal section of the enucleated globe (F). Disseminated melanoma cells are noted along the nasal retinal mass (a, 1; Melan-A, original magnification, ×100), optic nerve (b, 2; HMB-45, original magnification, ×40), fovea (c, 3; hematoxylin–eosin, original magnification, ×200), and perivascular distribution (d, 4–5; hematoxylin–eosin, original magnification, ×400 and ×200 respectively).

At postoperative week 2, the IOP was 35 mmHg in the left eye, raising concern for secondary melanomalytic glaucoma. The patient wished to avoid enucleation. Salvage therapy was attempted by injection of melphalan (20 μg/0.05 mL), 0.02 mL administered intracamerally and 0.03 mL intravitreally at postoperative months 1 and 2. On the succeeding month, a new episcleral brown lesion on the bulbar surface, iris bombe, and 4+ brown/pigmented cells in the anterior chamber were found. B-scan showed that the size of the posterior segment mass had increased. Owing to the significant progression, enucleation of the left eye was performed 7 months from the presentation. Magnetic resonance imaging (MRI) of the brain and PET/CT were negative. Histopathology of the enucleated globe revealed melanoma cells invading the trabecular meshwork, angle, and retina, optic nerve, and suture tracks, with perivascular spread (Figure 3). No disease recurrence was observed 6 weeks after enucleation. The patient was lost to follow-up.

### Case 3

**Figure 4.**
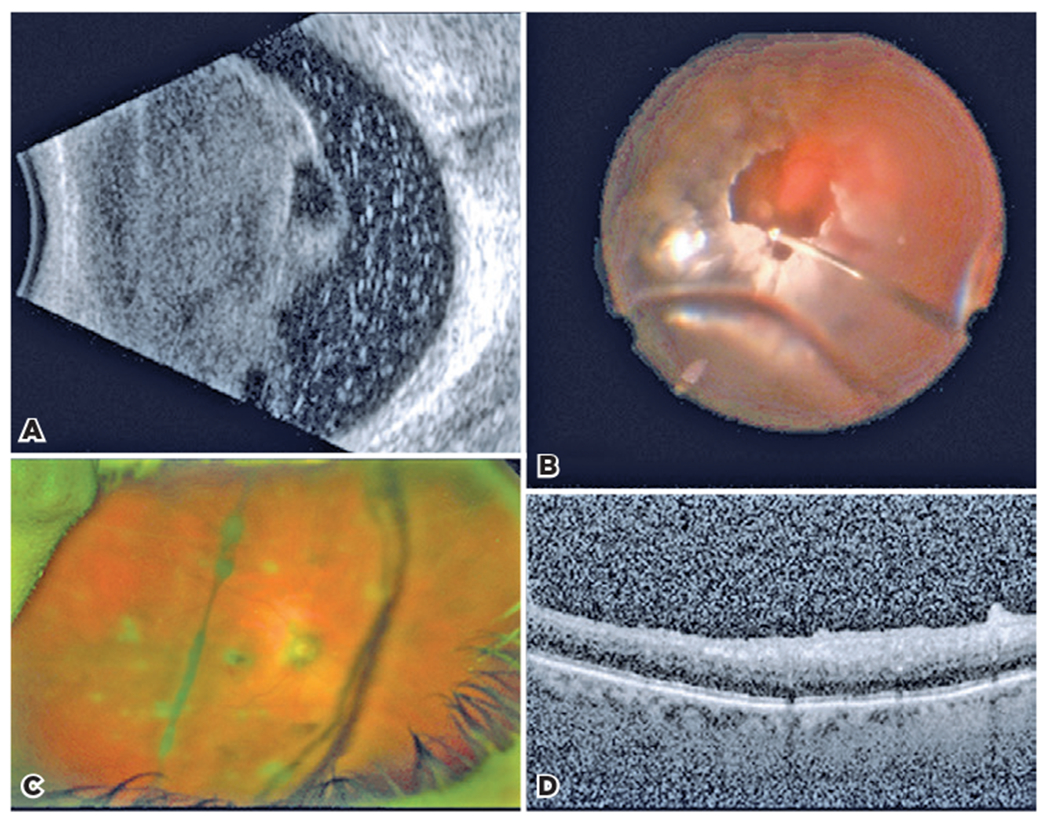
Cases 3 and 4 presented vitreous metastasis of cutaneous melanoma masquerading as intermediate uveitis. (A) B-scan initially showed dense vitreous debris limiting the view to the fundus. (B) Intraoperative image of the diagnostic vitrectomy in case 3 shows a predominance of amelanotic cells. There were clear media and absence of chorioretinal lesions postoperatively following vitrectomy and six intravitreal injections of melphalan. The post-diagnostic vitrectomy findings in case 4 included (C) few vitreous opacities and punched-out chorioretinal scars seen on wide-field fundus photo and (D) OCT demonstrating an irregular retinal surface with few deep vitreous cells.

A 68-year-old woman presented with vision loss, flashes, and floaters in the right eye for 2 months. The best-corrected VA (BCVA) was 20/800 in the right eye and 20/20 in the left eye. IOPs were 14 mmHg bilaterally. A slit lamp examination of the right eye revealed fine, brown keratic precipitates, 2-3+ anterior chamber cells, Koeppe nodules, and brown pigment granules adherent to the anterior and posterior capsules. The fundus examination was limited. B-scan ultrasonography showed vitreous opacities without masses (Figure 4A). Inflammatory and infectious labs were negative. Subtenon’s triamcinolone and a combination of topical steroids and cycloplegics were unsuccessful in controlling the assumed inflammation. The viral PCR of the anterior chamber was negative. Further investigation uncovered a personal history of facial and lower extremity CM, with CNS metastases 2 years preceding eye complaints. The patient was managed by surgical resection of the primary tumor, gamma knife, and radiation for CNS lesions and was still receiving pembrolizumab (Keytruda) at the time of ophthalmic presentation. Brain MRI 5 days before the ocular presentation was negative.

**Figure 5.**
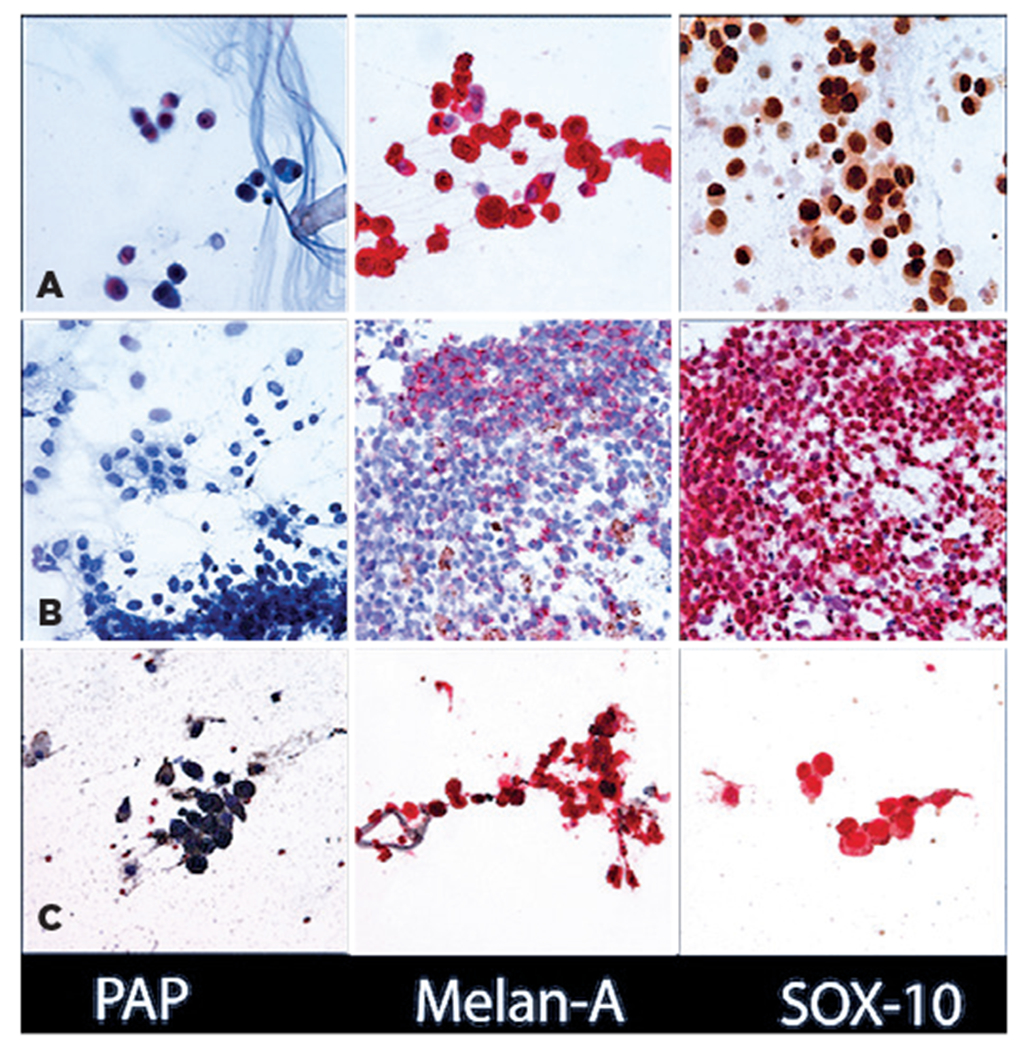
Cytology results of diagnostic vitrectomy in cases 3 (A), 4 (B), and 5 (C). From left to right: (A) PAP (original magnification, ×600), Melan-A with red chromogen (original magnification, ×600), and SOX-10 (×600). (B) PAP (original magnification, ×600), Melan-A with red chromogen (original magnification, ×600), SOX-10 with red chromogen (original magnification, ×600). (C) PAP (original magnification, ×600), Melan-A with red chromogen (original magnification, ×600), SOX-10 with red chromogen (original magnification, ×600).

A diagnostic PPV of the right eye was performed. Intraoperatively, vitreous opacities were predominantly amelanotic (Figure 4B). Cytology was consistent with melanoma (Figure 5A), masquerading as intermediate uveitis. PET/CT was negative for distant metastases. The patient underwent six monthly intravitreal injections of melphalan and continued topical steroids. At 10 months follow-up from baseline, BCVA was 20/40 and the IOP was 20 mmHg. Fundus examination showed trace anterior chamber cell and no chorioretinal lesions. MRI and whole body PET-CT 10 months later remained negative for systemic metastasis. Two months later, the patient developed a new CM lesion on her thigh and underwent treatment with 6 talimogene laherparepvec (Imlygic^®^, Amgen Inc., CA, USA) and 4 ipilimumab infusions with a good systemic response.

The patient developed a total retinal detachment in the right eye at 21 months follow-up from the initial presentation. She underwent complex retinal detachment repair with PPV, membrane peeling, silicone oil, and intravitreal injection of melphalan. The patient maintained good anatomical outcomes with hand-motion VA and no recurrence of VMCM at postoperative month 14 (36 months from the original VMCM presentation).

### Case 4

An 88-year-old woman with myelodysplastic syndrome and a history of CM without metastasis 15 years prior presented with 6 months of right eye floaters. A PPV elsewhere showed atypical melanoma cells. The patient was seen by our ocular oncology service in the third postoperative week. BCVA was 20/30 + 2 in the right eye and 20/25-2 in the left eye. IOPs were 35 and 16 mmHg in the right and left eyes, respectively. The anterior segment examination of the right eye revealed a posterior chamber intraocular lens with capsular opacification that was managed with YAG capsulotomy. Fundus examination was limited. B-scan ultrasonography showed a focal hyperechoic lesion attached to the vitreous skirt superonasal. IOP-lowering drops were initiated. She refused systemic workup of metastatic CM.

Twelve weeks later, VA declined to 20/125, and the vitreous was hazy with non-pigmented cells. Subtenons triamcinolone was administered. Two weeks later, vision improved to 20/80, but declined to 20/400 at 5 weeks post-injection. At that time, brown condensations were found within the vitreous and over the macula. Repeat diagnostic PPV confirmed VMCM (Figure 5A). Punchedout chorioretinal lesions were visualized, mimicking multifocal chorioretinitis (Figure 4C). Intravitreal chemotherapy was deferred due to high IOP and minimal melanotic debris. OCT at postoperative month 1 showed inactive preretinal cells (Figure 4D). The VA was 20/1000, and the IOP was 18 mmHg. There were inferior keratic precipitates, 1+ anterior chamber cell, and amelanotic material along lens surfaces and vitreous.

Melphalan was unavailable at the patient’s preferred clinic site; thus, intravitreal administration of methotrexate (400 μg/0.1 mL) was injected at weeks 1, 2, 6, and 10. VA was stable at 20/200, and the IOPs were 14-29 mmHg on topical IOP agents. The patient was lost to follow-up for 4 months during the COV1D-19 pandemic. She returned with eye pain, a 3-mm hyphema, and dense vitreous hemorrhage without visible NVI. VA was light perception [LP], and the retina was attached without a mass on B-scan. The IOP was 44 mmHg due to discontinued glaucoma therapy, which was restarted. Three months later, vision declined to NLP due to neovascular glaucoma (NVG). The patient remained comfortable with observation and topical IOP-lowering therapy 34 months after the initial presentation.

### Case 5

An 82-year-old woman 1 month of right eye vision loss and floaters was referred due to pigmented vitreous opacities noted on PPV for a presumed non-clearing vitreous hemorrhage. Examination at our facility revealed BCVA values of 20/100 in the right eye and 20/40 in the left eye. IOPs were 31 and 15 mmHg, respectively. A slit lamp examination showed brown deposits along the corneal endothelium, posterior capsule, and vitreous (Figure 1D). There was moderate nuclear sclerosis. The retina appeared flat with diffuse pigment on limited examination (Figure 1E). High-resolution ultrasonography and B-scan showed opacities along the residual vitreous skirt and cavity (Figure 1F).

The patient revealed a history of metastatic CM, treated with nivolumab 3 years ago. PET/CT scan was normal 1 month before the ocular presentation. The patient underwent cataract extraction, intraocular lens implantation, and PPV with intravitreal administration of melphalan. Cytology was consistent with metastatic melanoma (Figure 5C). Four weeks later, his BCVA was 20/60+1. At postoperative month 4, following three intravitreal injections of melphalan, VA was 20/400, and the IOP was 22 mmHg. Brown pigment deposits over the optic nerve and retinal surfaces markedly improved with serial injections (Figure 1G–J). The residual cells were felt to represent inactive melanoma. Inflammatory cells were treated with topical difluprednate. Secondary glaucoma with IOP of 31 mmHg led to cyclophotocoagulation. VA was counting fingers 22 months after the second PPV.

## DISCUSSION

To our knowledge, this study of five patients with VMCM is the largest single-institution report at present. There may be an increased likelihood of patients with CM developing VMCM in the era of CPI therapy because of improved patient survival and the idea that the eye is an immune-privileged site, resistant to CPI treatment^(5,9)^. Therefore, knowledge of the features of VMCM to allow for early diagnosis and management is increasingly important.

Sites of ocular metastasis of CM include the vitreous (most common), choroid, retina, iris, ciliary body, optic nerve, anterior chamber, and trabecular meshwork. Eyelid and orbit involvements are less common. The mechanism of ocular spread may include the CSF/optic nerve, pars plana, or hematogenous via permeable retinal vessels^(2,3,5,9)^. When suspected, VMCM can be confirmed by a vitreous/retinal biopsy or analysis of a whole-eye specimen. PAP and/or H&E stains demonstrate atypical basophilic cells with prominent nucleoli. Positive stains for Melan-A/MART-1, S-100, HMB-45, and a high Ki-67 proliferative index are consistent^(1,2,4,9)^.

Francis et al. published a multicenter, retrospective cohort study of 14 eyes of 11 patients with VMCM. The authors showed a histological image of diffuse pigment along the blood vessels^(9)^. Similarly, case 2 showed melanoma cells invading the optic nerve and perivascular and intravascular spaces (Figure 3). This supports hypotheses regarding the CNS and the hematogenous mechanism of the ocular entry of CM cells. Interestingly, case 2 had evidence of trans-scleral invasion through the sutured sclerotomy site (Figure 3D). This suggests an iatrogenic mechanism of CM spread and highlights the importance of adequate cryotherapy following sclerotomy closure after diagnostic PPV. While Francis et al. noted concomitant CNS metastasis of CM in patients with optic nerve invasion^(9)^, this was not seen in our cohort.

For cases in which the distinction between VMCM and PUM is unclear, genetic markers may be explored as a diagnostic tool. CMs carry mutations in *BRAF, NRAS, CDKN2A*, and *PTEN*. PUMs have mutations in *GNAQ/GNA11* and may lose *BAP1* and other tumor suppressor genes (*CDKN2A* and *PTEN*). PUM may be associated with monosomy 3 and lack BRAF mutations^(10,13)^. Immunohistochemical stains such as CD68 and HMB45 were useful in diagnosing our patients. None of our cases required genetic analysis to differentiate VMCM and PUM due to a known history of CM. Interestingly, case 2 masqueraded as a biconvex posterior mass; genetics could have been considered if the diagnosis was unclear.

As described by Francis et al.^(9)^, nearly half (2/5) of our cases presented with amelanotic vitreous opacities rather than a “pigmented vitritis.” Amelanotic VMCM masquerading as posterior or intermediate uveitis, as in cases 3 and 4, should be kept on the differentials in patients with a history of CM. Early diagnosis requires high clinical suspicion and a low threshold for a diagnostic PPV in such cases.

Treatment approaches vary widely across reports of metastatic ocular CM, including external beam radiation, debulking PPV, and enucleation. Systemic chemotherapy is inadequate for intraocular tumor control^(3,5)^. Intravitreal administration of melphalan was described by Francis et al. as an effective treatment in VMCM^(9)^. Intravitreal administration of melphalan is also used to treat ocular tumors such as retinoblastoma (20-30 μg/mL), vitreoretinal lymphoma (10 μg/mL), and PUM with pigment dissemination (various doses). Its use may be limited by retinal toxicity^(9,14,15)^. Intravitreal administration of methotrexate (400 μg/0.1 mL) is less toxic to the retina and is used in PUM but not in VMCM^(14)^. Three of the five patients in our cohort received intravitreal administration of melphalan without retinal toxicity. Case 4 received intravitreal administration of methotrexate because of accessibility; vision remained stable until being lost to follow-up for 4 months. This is the first study to describe intravitreal administration of methotrexate to stabilize VMCM, but further study is indicated to determine its efficacy.

Historically, most eyes with VMCM were enucleated because of the high rates of ocular invasion and NVG. However, no evidence suggests that enucleation prolongs survival^(1,5,9,10)^. Only one of the five patients in our cohort underwent enucleation due to a progressive ocular spread of melanoma cells. Francis et al. reported that 1/17 eyes required enucleation in the absence of intravitreal administration of melphalan. In 2007, before intravitreal chemotherapy for VMCM, Jaissle et al. reported that in 22 eyes of 17 patients, 6 eyes required enucleation^(4)^. Further study is needed to determine if intravitreal administration of melphalan reduces the risk of progressive VMCM requiring enucleation.

The recommended dosing, frequency, and duration of intravitreal administration of melphalan in treating VMCM remain unclear. Paez-Escamilla et al. reported the efficacy of melphalan as a single injection (32 ug/0.075 mL) following five injections of methotrexate (400 μg/0.1 mL) in treating PUM with vitreous involvement^(14)^. Francis et al. reported intravitreal administration of melphalan 10-20 μg monthly for 1-5 total doses. They noted that 2/3 of the eyes receiving single treatments with melphalan (10 μg/0.05 mL) responded, whereas 1/3 eye had disease progression with four monthly doses of 20 μg. They contended that increasing the dose to 20-30 μg, as in retinoblastoma, may provide additional effects^(9)^. Our series showed a reduction in tumor burden with intravitreal administration of melphalan 32 μg/0.075 mL monthly for 4-6 doses, including at the time of PPV in patients with very high suspicion.

The therapeutic endpoint of VMCM and PUM with vitreous dissemination remains unclear. In agreement with Metz et al., not all cells are vital melanoma cells. Reassessing the presence of melanoma cells by obtaining a vitreous specimen may help differentiate them from melanophages^(14,16)^. Practically, this was not believed to be necessary in the present cohort. Serial photographs looking for interval cellular proliferation during a break from intravitreal chemotherapy helped establish a treatment endpoint in case 5 (Figure 1G–J).

Increased awareness and earlier detection of VMCM in the CPI era is important, as an earlier intervention with PPV and intravitreal chemotherapy may improve ocular and visual outcomes.

## Figures and Tables

**Table 1.** Demographics and clinical characteristics of ophthalmic findings in our patients with vitreous metastasis of cutaneous melanoma

	Case 1	Case 2	Case 3	Case 4	Case 5
**Age (years) at Presentation**	37	87	68	88	82
**Sex**	Male	Male	Female	Female	Female
**Duration of Symptom(s)**	2 days	3 months (1 month worsening)	2 months	6 months	1 month
**Laterality of Disease**	Right	Left	Right	Right	Right
**Presenting Symptom(s)**	Vision loss Eye pain	Vision loss Floaters	Vision loss Floaters Flashes	Vision loss Floaters	Vision loss Floaters Referral(s/p PPV 3 months prior)
**Presenting Sign(s)**	NVG Hyphema	Vitreous hemorrhage	Anterior uveitis Intermediate uveitis	Pigment on the IOL capsule	Vitreous hemorrhage (Recurrent, with pigment)
**Initial Examination Findings**	Posterior synechiae Corneal edema No view of fundus	Cataract Pigment on capsule No view of fundus	Fine pigmented KP 2-3+ AC cell Koeppe nodules Pigment on capsule No view of fundus	Opacification of the IOL No view of the fundus	Cataract Pigment on the capsule Hazy view of the fundus Retina flat and pigment changes
**B-scan ultrasound (Presentation)**	Vitreous opacities Membrane No masses	Vitreous opacities Vascularized domeshaped mass 9:30 (7.5r × 7.5c × 1.3 mm)	Vitreous opacities No masses	Focal hyperechoic source (attached to vitreous skirt) No marked vitreous cells	Vitreous opacities Opacity on the vitreous skirt No masses
**Intraoperative Findings (Additional)**	CRVO (Diffuse DBH and CWS)	Retinal tear Pigmented ERM Retinal mass (nasal)	“Chalk-white” vitreous No focal retinal lesions	Multifocal chorioretinal lesions	Diffuse and perivascular pigmented deposits in the fundus
**Melanin Status**	Melanotic	Melanotic	Amelanotic	Amelanotic/melanotic	Melanotic
**VA (Initial)**	20/100 (uncorrected)	Hand motions	20/800 (BCVA)	20/30+2 (BCVA)	20/100 (BCVA)
**VA (Final)**	Not available	Enucleated	20/40 (BCVA) pre-RD HM (post-RD)	20/1000 (BCVA) pre-NVG NLP (post-NVG)	20/CF (BCVA)
**Intraocular Pressure (Initial)**	22	13	14	14	31
**Intraocular Pressure (Final)**	Not available	35	20	20	22
**Ocular Treatments**	1. Intravitreal bevacizumab 2. Phaco/IOL/PPV/Vit biopsy/IV triamcinolone 3. Unavailable thereafter	1. PPV/Vit biopsy 2. PPV/MP/EL/AFx 3. Melphalan × 2 (rescue) 4. Enucleation	1. Subtenons triamcinolone 2. PPV/Vit biopsy 3. Melphalan × 6 (monthly) 4. PPV/MP/Oil/Melphalan for complex RD repair	1. PPV/Vit biopsy (outside) 2. Nd:YAG capsulotomy 3. Glaucoma medications 4. PPV/Vit biopsy 5. Methotrexate ×4 (weeks 1, 2, 6, and 10) for amelanotic globules	1. PPV/Vit biopsy (outside) 2. Phaco/IOL/PPV/Vit biopsy/Melphalan 3. Melphalan × 3 (monthly)
**Response to Ocular Treatments**	Not available	Poor (iris bombe, episcleral pigment 6 weeks after the first melphalan injection)	Good	Stabilized until lost to follow-up	Good
**Follow-up (Months) from DX**	Not available	8.5	36	34	22
**Final Ocular Disease Status**	Not available	Enucleated due to NVG	No melanoma cells Pigment on the lens surface No glaucoma	Amelanotic globules/haze (lens capsule and retinal surface) NVG after lost to follow-up	Inactive pigmented cells Secondary glaucoma

AC= anterior chamber; CF= counting fingers; CRVO= central retinal vein occlusion; CWS= cotton wool spots; DBH= dot blot hemorrhages; DX= diagnosis; IOL= intraocular lens; MP= membrane peel; Nd:YAG= neodymium-doped yttrium aluminum garnet; NVG= neovascular glaucoma; NVG= neovascular glaucoma; Phaco= phacoemulsification; PPV= pars plana vitrectomy; RD= retinal detachment; Vit= vitreous.

**Table 2.** Clinical characteristics of the systemic findings in our patients with vitreous metastasis of cutaneous melanoma

	Case 1	Case 2	Case 3	Case 4	Case 5
**Prior Systemic Comorbidities**	Healthy	Healthy	Healthy	Myelodysplastic syndrome	Healthy
**Time of Skin Melanoma**	2 years prior	<2 years prior	2 years prior	15 years prior	3 years prior
**Year of Ocular Diagnosis**	2007	2017	2019	2019	2019
**Site of Skin Melanoma**	Right shoulder	Right cheek	Right thigh Head and neck	Left arm	Unknown
**Metastasis**	Lymph nodes Axilla Chest wall	Lymph nodes Parotid gland	CNS (brain and spine)	Unknown (Patient refused imaging)	Unknown (Not available)
**Prior Systemic Treatment**	Surgical resection Systemic interferon (subcutaneous, intravenous) External beam radiation (3600 Gy)	Mohs surgery and right cheek Nivolumab	Chemotherapy Gamma knife External beam radiation Pembrolizumab	Surgical resection only No systemic therapy	Nivolumab
**Systemic Treatment (Current)**	Interferon	None(Last dose 3 weeks prior)	Pembrolizumab	No	None
**Systemic Disease Status (by whole body imaging)**	Active at the time of eye DX	Remission	Inactive/controlled New cutaneous melanoma lesion	Unknown, clinically well (Patient refused imaging and denies non-ocular symptoms)	Remission
**Vital Status**	Unknown (Unavailable)	Unknown (Lost to follow-up)	Alive	Alive	Alive

CNS= central nervous system; DX= diagnosis.
